# 

**Published:** 1890-08-02

**Authors:** 


PRICE ONE PENNY.

[No.] 201, Vol. VIII.—August 2nd, 1890.

[Re]gistered at the General Post Office as a Newspaper for transmission in the United Kingdom and Abroad.]

WITH EXTRA NURSING SUPPLEMENT.
Per Annum, 6s. 6d.; post free.
Monthly Parts, 6d.; post free 8d.
Ditto per Annum, 8s., post free.

### THE HOSPITAL

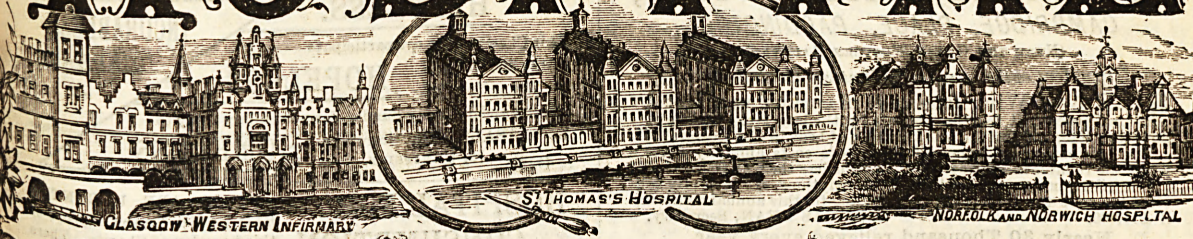

A WEEKLY JOURNAL OF

### Science, Medicine, Nursing and Philanthropy.

SATURDAY, AUGUST 2nd, 1890.

